# Allergic/infusion reactions reported with cetuximab and rituximab

**DOI:** 10.1186/1939-4551-8-S1-A82

**Published:** 2015-04-08

**Authors:** Ray A Wolf, Mary-Ellen Marx, Laura Szalaj, Jeffrey J Kuper, Paul F Cavanaugh

**Affiliations:** 1 Mylan Specialty L.P., USA; 2 PharmaWrite, LLC, USA

## Background

Monoclonal antibodies (mAbs) used in cancer therapy may cause allergic/infusion reactions (AIRs). To assess the scope of this problem, a pilot literature search was conducted.

## Methods

Using terms for oncology mAbs and AIRs, English-only articles were searched in PubMed, EMBASE, and BIOSIS. The search was conducted on Nov 19, 2013, with no date limiters. Cetuximab (C) and rituximab (R) were among the most commonly reported mAbs, and these were analyzed further. Articles were excluded if AIRs couldn’t be quantified.

## Results

940 articles met criteria, including 167 and 83 for C and R, respectively, describing 19,861 and 5064 mAb-treated patients. AIRs were reported in 1694 (8.5%) and 647 (12.3%) C and R patients, but these numbers may be low as some articles only reported grade 3/4 or severe reactions. Various terms were used to describe AIRs (eg, anaphylaxis [AX], allergic reaction [AR], hypersensitivity reaction [HR], infusion reaction [IR]), so it was often difficult to distinguish the type of AIR. Some AIRs were reported as grade 4 HRs but not specifically noted as AX. Also, it was hard to attribute an AIR to a specific agent when the mAb was part of combination therapy. Grade 3/4 AIRs were noted in 633 (3.2%) and 215 (4.2%) C and R patients. AX specifically was noted in 88 (0.4%) and 64 (1.3%) C and R patients. Among C patients, 13 fatalities were attributed to AIRs, with none in the R group.

## Conclusions

Although a well-known adverse event, the magnitude of AIRs with C and R still may be underappreciated. The presence of the galactose-galactose epitope in mAbs produced in murine cell lines, as with C, contributes to AIRs. The frequency of AIRs was likely underestimated in this review due to limited reporting in the medical literature and varied classification. Various AIR terms were used interchangeably, sometimes within a single article. Grade 4 AIRs were not always labeled as AX. Grade 3/4, AX, or fatal AIRs were rare but are clinically significant when they occur. Management of AIRs often was not reported, nor was the incidence of delayed AIRs, which may have been unknown. This suggests a need for more standardized reporting of AIRs. Appropriate steps for prevention/treatment of AIRs should be taken when these mAbs are utilized. Readily available therapies for the treatment of AIRs may be helpful (eg, epinephrine autoinjectors); however, these products are not indicated for patients at risk for delayed AIRs.

